# Can red deer antlers be used as an indicator of environmental and edible tissues’ trace element contamination?

**DOI:** 10.1007/s11356-017-8798-7

**Published:** 2017-03-21

**Authors:** Aleksandra Giżejewska, Józef Szkoda, Agnieszka Nawrocka, Jan Żmudzki, Zygmunt Giżejewski

**Affiliations:** 1 Department of Pharmacology and Toxicology, Faculty of Veterinary Medicine, University of Warmia and Mazury, Oczapowskiego 14, 10-719 Olsztyn, Poland; 2 The National Veterinary Research Institute, 57 Partyzantów Avenue, 24-100 Puławy, Poland; 3 Institute of Animal Reproduction and Food Research of Polish Academy of Sciences, Tuwima 10 Str, 10-748 Olsztyn, Poland

**Keywords:** Toxic trace elements, Essential trace elements, Bioindicator, Pollution

## Abstract

Venison is an attractive product for consumers concerned with healthy lifestyle; however, it can contain high levels of toxic elements, and therefore, it is a possible source of hazardous contaminants in human diet. Antlers are suitable bioindicators of environmental metal contamination, and herein, we assessed the ability of trace element levels in antlers to indicate levels in edible soft tissues. We determined the concentrations of lead (Pb), cadmium (Cd), mercury (Hg), arsenic (As), copper (Cu), zinc (Zn), and iron (Fe) in the liver, kidney, muscle, and antlers of 14 free-ranging red deer (*Cervus elaphus*) from northeastern Poland using atomic absorption spectrometry. We found the highest concentrations of Pb (0.321 ± 0.165 mg/kg), As (0.045 ± 0.074 mg/kg), Zn (105.31 ± 16.33 mg/kg), and Fe (220.92 ± 117.18 mg/kg) in antlers; of Cd (4.974 ± 1.90 mg/kg) and Hg (0.048 ± 0.102 mg/kg) in kidney; and of Cu (7.29 ± 7.02 mg/kg) in the liver. A positive relationship between concentrations in antlers and muscle was found only for Cu (*p* = 0.001), and it therefore appears that red deer antlers cannot be used as an index for element concentrations in soft tissues. While our results confirm that the Mazury region is little polluted, consumption of red deer offal from this area should be limited according to extant legal limits set for livestock consumption.

**Electronic supplementary material:**

The online version of this article (doi:10.1007/s11356-017-8798-7) contains supplementary material, which is available to authorized users.

## Introduction

Despite modern technologies of exhaust gas purification, sewage treatment, and improvement of mining and metal processing methods, contaminants deposited in the environment in recent years remain of major concerns (EFSA 2009, 2010; Küttner et al. 2014). Toxic trace elements are present not only in the vicinity of industrial areas but also in natural and agricultural ecosystems away from emission sources (Giżejewska et al. 2015). Toxic trace elements such as lead (Pb), cadmium (Cd), mercury (Hg), and arsenic (As) pose a health threat to organisms even in small doses. Although essential trace elements such as copper (Cu), zinc (Zn), or iron (Fe) are necessary for growth and development of organisms, they may also pose a threat in high doses (Wasi et al. 2013).

Venison is an attractive product for consumers due to its nutritional properties, i.e., high protein and essential element content, optimal fatty acids, and low fat contents (Skibniewski et al. 2015). Most popular game species consumed in Europe are wild boar (*Sus scrofa*), roe deer (*Capreolus capreolus*), and red deer (*Cervus elaphus*; Falandysz et al. 2005; Ramanzin et al. 2010). In countries with hunting tradition, venison constitutes a large percentage of the annual meat consumption, and in Poland it was assessed as 80 g per capita per annum on average (Górecka and Szymańko 2010). In hunters’ families, however, venison consumption can be much higher (e.g., 1–4 kg per capita per annum in Italy; Ramanzin et al. 2010). Edible tissues of free-living animals can contain higher levels of toxic trace elements than livestock and could therefore potentially be an important source of hazardous contaminants in human diet (Kramárová et al. 2005). Moreover, while maximum levels of toxic trace elements in animal products are set by the European Committee (EC) for livestock (bovine, sheep, pig, and poultry), there are no legal limits set for venison.

Liver and kidney being the most important organs responsible for detoxification, they most often are tissues of choice in analyses of environmental pollution levels (Stankovic et al. 2014). Contrastingly, cervid antlers have a number of advantages that make them a unique material for monitoring of trace contaminants (Giżejewska 2015; Giżejewska et al. 2016, 2015). Antlers are bony cephalic appendages that are cast and regenerated annually, with high demand for minerals in the growing period, during which they accumulate trace elements (Kierdorf and Kierdorf 2006). Antlers suitability for bioindication of current and retrospective environmental contamination by trace elements (Giżejewska 2015), fluoride (Kierdorf and Kierdorf 2006), and radionuclides (Baeza et al. 2011) has been demonstrated. Moreover, antlers are readily available when collected either as trophies during scheduled hunts or non-invasively when naturally cast antlers are recovered.

Red deer has many features of a good bioindicator species as (i) its biology is relatively well known; (ii) it has a relatively long life span; (iii) it is widespread in temperate latitudes, relatively sedentary, and can come close to human settlements; and (iv) it has a defined diet. If content of elements in red deer soft tissues could be predicted based on their levels in antlers, chemical analysis of antler composition would allow health risk assessment for consumers of red deer offal and meat. To our knowledge, there is no study assessing the relationships between trace element concentrations in red deer antlers and their levels in soft tissues. To address this research gap, (i) we determined the concentrations of Pb, Cd, Hg, As, Zn, Cu, and Fe in the liver, kidney, muscle tissue, and antlers of red deer hunted in an area regarded as little contaminated; (ii) we assessed the ability of trace element levels in antlers to indicate levels in edible soft tissues; and (iii) we evaluated the degree of contamination of red deer edible tissues, according to the EC limits set for livestock, with regard to the lack of an official threshold for wildlife.

## Materials and methods

### Study area

We investigated the levels of trace elements in red deer hunted in the Pisz Forest, Warmia and Mazury Region, north-eastern (NE) Poland (21° 32′ E, 53° 40′ N; ca. 170 km^2^). The region is a traditional agricultural area with extensive continental and sub-continental mixed coniferous forests (31% of the area), and numerous lakes. The region is an important recreational center for local and international tourists who seek activities in a natural environment. Pisz Forest is the largest forest complex in Mazury and the second largest in Europe. The sampling site was away from any major industrial complex as putative source of pollutants (Giżejewska et al. 2015) but was 20–30 km from a military training ground in Orzysz.

### Sampling

We used tissue samples from 14 red deer stags culled in the hunting season 2013/2014 in accordance with current hunting plans and regulations. Age of the animals was from 4 to 11 years and was assessed based on teeth wear (Lowe 1967) and body weight. We collected ca. 300 g of liver, kidney (contain renal cortex and medulla), muscle (medial side of quadriceps femoris), and antler tissues from each individual. We sampled antlers using rechargeable hand drill fitted with vanadium drill (6-mm diameter). Before sampling, antlers were carefully cleaned with a nylon brush. Afterwards, a hole was drilled into the back of the beam approximately 6 cm into the brown tine (the first antler branch), 1.5 cm above the burr. Samples contained both compact and trabecular antler bone. To avoid secondary contamination of samples, the drill was rinsed in distilled water after each sample was collected. All samples were placed in separate polyethylene bags, then were frozen, and stored at −20 °C until analysis.

### Chemical analysis

Homogenized samples were weighed in duplicate (0.5–10 g) and deposited in quartz crucibles, oven-dried overnight (120 ± 20 °C), and then ashed in a muffle furnace at 450 °C for 24 h (550 °C for As). All chemicals were of analytical grade. Concentrated nitric acid was added (1 ml) to ashed samples, evaporated on a hot plate, and ashed again in the muffle furnace for about 1 h. This last step was repeated until we obtained carbon-free ash (usually three times). In sample preparation for As analysis, 50% *v*/*v* magnesium nitrate hexahydrate solution was added. Before analysis, samples were diluted in 0.2% *v*/*v* nitric acid (for Pb and Cd) or 1 N HCl (for other elements). We performed graphite furnace atomic absorption spectrometry to determine Cd and Pb concentrations using atomic absorption spectrometer Perkin-Elmer 4110 ZL (Szkoda and Żmudzki 2005). We used flame atomic absorption spectrometry with atomization in acetylene/air flame to determine Cu, Zn, and Fe concentrations using AVANTA PM (GBC, USA) spectrometer. Arsenic concentration was determined by hydride generation atomic absorption spectrometer PinAAcle 900T equipped in electrodeless discharge lamp (As EDL) and flow injection system FIAS 100 (PerkinElmer; Szkoda et al. 2006a). Concentrations of total Hg were determined using Advanced Mercury Analyzer AMA-254 (Altec Ltd., Czech Republic) performing atomic absorption spectrometry method, without prior sample mineralization (Szkoda et al. 2006b).

Analysis for each element was performed based on calibration curves plotted from blanks and working standard solutions. For calibration, we used commercial stock solutions (1000 μg/ml) of analytical grade for all four elements (J.T. Baker®). Calibration working standard solutions were 1–10 μg/l for Cd, 10–60 μg/l for Pb, 3–25 μg/l for As, 0.2–3.0 μg/ml for Cu, 0.1–0.7 μg/ml for Zn, and 0.5–3.0 μg/ml for Fe. For Hg calibration, two ranges of working solution were made, which are 0.05–0.50 and 1.0–5.0 μg/ml. Limit of detection (LOD) and limit of quantitation (LOQ) were calculated as 3 and 10 times SD from results of blank matrix measurements (*n* = 10), respectively, with a low analyte content. LOD was established at the following levels (mg/kg): for Cd and Hg 0.001, Pb and As 0.002, Cu 0.04, Zn 0.12, and Fe 0.11. Reagent, calibration blanks, and control samples were prepared and run in triplicate. Calibration was periodically verified by analyzing a standard every 20th reading.

Accuracy and precision of the method were confirmed by the analysis of certified reference materials (CRMs), bovine liver (BCR-185R, IRRM, Belgium), pig kidney (BCR-186, IRRM, Belgium), lobster *hepatopancreas* (TORT-2, NRC-CNRC, Canada), and compound feed (IMEP-117, IRRM, Belgium). Recoveries of analyzed elements were within the range of 96–110%, and relative standard deviations were below 10%. We expressed element concentrations in milligrams per kilogram of wet weight (mg/kg w.w.).

### Statistical analysis

For statistical analyses, concentrations below the LOD were assigned a value of half the LOD for each element (Reglero et al. 2008, 2009). To assess if concentrations in antlers could be used as an index of concentrations in soft tissues, we used linear regression of concentrations of a given trace element in a given soft tissue against its concentration in antlers. Because trace elements could bioaccumulate with age (López Alonso et al. 2004), we tested for additive and interactive effects of the age of the individuals. No age effect was detected and we report the results of univariate regression against concentration values in antlers. Statistical significance was set at *p* < 0.05. All trace element concentration data were natural log-transformed to meet statistical assumptions. We conducted all statistical analyses using R version 3.0.0 (R Core Team 2013).

## Results

Summary statistics of the concentrations of the seven trace elements analyzed in the four tissues are reported in Table 1. In the muscle tissue, Pb concentration in four samples (0.082, 0.119, 0.079, and 0.570 mg/kg) were higher than in the liver samples of the corresponding individuals (0.020, 0.015, 0.027, and 0.016 mg/kg, respectively). These four muscle samples were excluded from the regression analysis against concentrations of Pb in antlers.

**Table 1:** Mean concentrations and range (mg/kg w.w.) of seven elements in four tissues of red deer, NE Poland, 2013–2014

Element	Number	Liver		Kidney		Muscle		Antler	
		Mean ± SD	Range	Mean ± SD	Range	Mean ± SD	Range	Mean ± SD	Range
Pb	14	0.043 ± 0.025	0.012–0.103	0.062 ± 0.024	0.034–0.109	0.079 ± 0.016	0.006–0.570	0.321 ± 0.165	0.093–0.649
Cd	14	0.256 ± 0.089	0.163–0.408	4.974 ± 1.90	1.311–6.925	0.006 ± 0.003	0.003–0.012	0.011 ± 0.004	0.006–0.021
Hg	14	0.007 ± 0.014	<0.001–0.048	0.048 ± 0.102	0.002–0.338	0.001 ± 0.000	<0.001–0.002	0.001 ± 0.002	<0.001–0.008
As	14	0.002 ± 0.001	<0.002–0.005	0.003 ± 0.002	<0.002–0.007	0.002 ± 0.002	<0.002–0.007	0.045 ± 0.074	<0.002–0.237
Cu	14	7.29 ± 7.02	1.87–27.05	4.08 ± 0.45	2.99–4.88	1.10 ± 0.35	0.50–2.09	3.14 ± 0.86	2.44–5.49
Zn	14	16.05 ± 3.44	11.01–23.27	52.64 ± 23.93	26.31–92.46	67.14 ± 15.67	45.15–94.18	105.31 ± 16.33	85.79–133.72
Fe	14	111.58 ± 50.59	34.08–195.16	75.93 ± 52.31	9.02–188.99	63.45 ± 35.62	22.49–152.53	220.92 ± 171.18	41.77–594.72

**Fig. 1:**
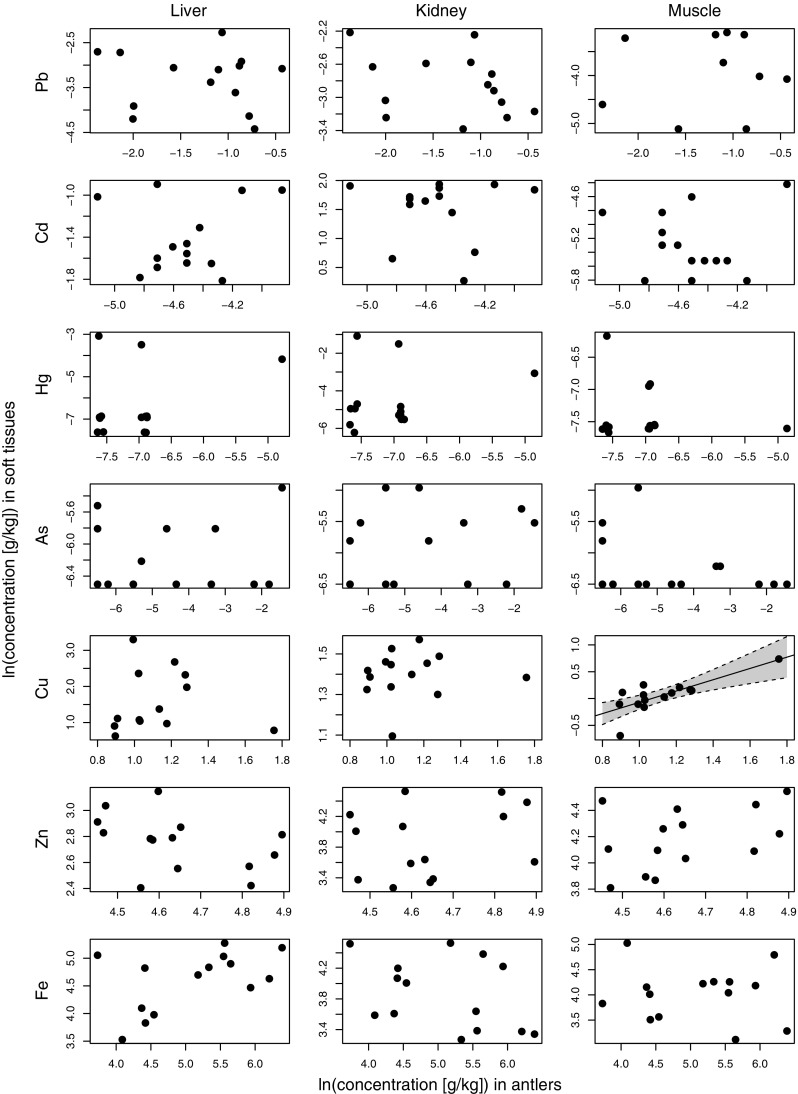
Concentrations of the Pb, Cd, Hg, As, Cu, Zn, and Fe in three edible soft tissues of red deer against concentration in the antlers of 14 individuals hunted in Mazury, NE Poland, 2013/2014. The only significant relationship was between antler and muscle tissue concentration for Cu. The *solid* represent the linear regression line, with the *dotted line* and *shaded area* depicting the 95% confidence interval. Note that all data have been ln-transformed to meet statistical assumptions. Four muscle tissue samples likely contaminated by ammunition have been excluded of the analysis of Pb in muscle versus antlers

Simple linear regression to assess the use of trace element concentrations in antlers as an indicator of soft tissue contamination showed only a significant, positive relationship between Cu concentrations in antler and muscle tissue (*F*_1,12_ = 18.48, *p* = 0.001, *R* ^2^ = 0.61; Fig. 1 and Table S1). Details of all other negative results are reported in Table S1.

Comparing concentrations of the four toxic trace elements to the maximum levels set by the European Commission for livestock, we found levels exceeding the norm in 2 muscle samples for Pb, 14 (100%) kidney samples for Cd, and 3 liver and 3 kidney samples for Hg (Table 2).

**Table 2:** Comparison between element levels in the analyzed samples of red deer tissues from Mazury, NE Poland, 2013/2014, and the legal categories for each metal according the European Commission (EC 2006, 2008)

Maximum levels (MLs) in tissues (mg/kg)	*n*	<LOD	P5	Median	P95	>ML
Pb						
Liver 0.5^a^	14	–	0.014	0.046	0.080	–
Kidney 0.5^a^	14	–	0.037	0.056	0.107	–
Muscle 0.1^a^	14	–	0.006	0.021	0.277	2
Antler	14	–	0.110	0.339	0.540	–
Cd						
Liver 0.5^a^	14	–	0.170	0.218	0.390	–
Kidney 1.0^a^	14	–	1.700	5.483	6.900	14
Muscle 0.05^a^	14	–	0.003	0.005	0.011	–
Antler	14	–	0.007	0.011	0.018	–
Hg						
Liver 0.01^b^	14	4	<0.001	0.001	0.036	3
Kidney 0.01^b^	14	–	0.003	0.007	0.026	3
Muscle 0.01^b^	14	11	<0.001	0.001	0.001	–
Antler	14	6	<0.001	0.001	0.003	–
As						
Liver	14	8	0.002	0.002	0.004	–
Kidney	14	6	0.002	0.003	0.007	–
Muscle	14	9	0.002	0.002	0.005	–
Antler	14	3	0.002	0.008	0.191	–

*n* number of samples, *P5* 5th percentile, *P95* 95th percentile, *<LOD* number of samples under limit of detection, *>ML* number of samples over the maximum legal limit

^a^EC 2006

^b^EC 2008

## Discussion

We tested whether antler chemical composition could be used as an index of trace elements in edible, soft tissues. The only significant relationship we found was between muscle tissue and antlers for Cu. Therefore, it seems that only analyzing trace elements in antlers—that can be non-invasively collected once red deer naturally cast them annually—cannot be used as an indicator of trace element contaminations in edible tissues. Moreover, although animals for this study were sampled in a forest-dominated area, which derives considerable economic benefits from hunting, and considered to be little contaminated (Zalewski and Pacholska 2010; Giżejewska et al. 2015), we found several samples of soft tissues exceeding recommended levels for livestock consumption.

### Toxic trace elements

#### Pb

High Pb concentrations in antlers reflect its metabolism and chemical similarity to calcium, which causes 90% total body burden of Pb to accumulate in bone tissues as phosphates (Reglero et al. 2009). Antlers, due to their annual complete regrowth and high demand for minerals, therefore accumulate relatively high levels of Pb.

In our study, Pb levels in the liver and kidney were low. Previous research conducted in NE Poland showed higher mean Pb levels in the liver, kidney, and muscle in both free-living (0.26, 0.36, 0.21 mg/kg; Falandysz et al. 2005) and farmed (0.700, 0.384, 0.388 mg/kg; Drozd and Karpinski 1997) red deer, whereas in more recent studies, Pb values were in the same order of magnitude than ours (0.17, 0.30, 0.18 mg/kg d.w. (Jarzyńska and Falandysz 2011); 0.10 mg/kg in muscles (Skibniewski et al. 2015)). This probably reflects the decrease of Pb levels in this region over time (Giżejewska 2015). Conversely, higher liver and kidney Pb concentrations were detected in red deer from mining (0.430, 0.805 mg/kg d.w.; Reglero et al. 2008, 2009) and hunting areas (0.57, 0.33 mg/kg; Santiago et al. 1998) in Spain, historic Cu-Ni-Fe ore smelting in Ontario (1.47, 1.95 mg/kg; Parker and Hamr 2001), and hunting areas close to industrial centers in Slovakia (1.904, 0.561 mg/kg; Kramárová et al. 2005). Data obtained in red deer from agricultural areas in Croatia (Lazarus et al. 2008; Bilandžić et al. 2009; Srebočan et al. 2012) were in the same order of magnitude as ours. Aerial borne particles are deposited on plant surface, and ingestion of contaminated vegetation represents the main source of Pb in herbivore (Srebočan et al. 2012). Additional sources are accidently or intentionally eaten soil and direct inhalation (Sheppard 2013).

In two muscle samples, we detected that Pb level exceeded the maximum threshold of 0.1 mg/kg recommended in farm animal edible tissues (Table 2; EC 2006). In four samples, Pb concentration was higher than in the liver, which likely indicated secondary contamination from Pb contained in ammunition (Szkoda et al. 2012). Against this background, these four samples were therefore excluded from the regression analysis while assessing the use of trace element concentrations in antlers as an index of concentration in muscle tissue. It has been shown that red deer muscle can be contaminated with Pb in the 30-cm neighboring area of the bullet pathway (Dobrowolska and Melosik 2008). In human, a diet including venison with high Pb concentration can result up to 2.5 times higher Pb exposure to the consumer, when we compare it to the exposure of human living on normal diet (EFSA 2010).

#### Cd

Cadmium, as well as Zn, Cu, or Hg, can induce the metallothionein (MT) synthesis and is transported to kidneys as Cd-MT complex. In our study, the recommended maximum 1.0 mg/kg Cd limit (EC 2006) was exceeded in all 14 kidney samples (Table 2). In study conducted in another little contaminated area, this limit was exceeded in 68% of roe deer and 84% of red deer samples (Wieczorek-Dąbrowska et al. 2013).

Previous research conducted in NE Poland reported liver and kidney Cd levels of respectively 0.23, 2.70 mg/kg (Falandysz et al. 2005) and 0.70, 12.0 mg/kg d.w. (Jarzyńska and Falandysz 2011). This indicates a stable level of this element in red deer habitat in the Masurian region. Conversely, Cd concentrations in farmed deer were much lower (liver 0.039, kidney 0.434, muscle 0.005 mg/kg; Drozd and Karpinski 1997), indicating the importance of dietary exposure; livestock do not eat perennial plants, which can accumulate higher contents of toxic elements.

Higher Cd contents in both liver and kidney were observed near an ore smelter in Ontario (2.44, 24.64 mg/kg; Parker and Hamr 2001). Surprisingly, higher liver Cd levels were found in our study than in mining areas in Spain (Reglero et al. 2008, 2009), Poland (Szkoda et al. 2012), and Croatia (Lazarus et al. 2005, 2008; Bilandžić et al. 2009). Kidney Cd concentrations found in red deer in Slovakia (2.387 mg/kg; Gasparik et al. 2004) and in Spain (2.16 mg/kg; Santiago et al. 1998) were twice lower than our results. In the liver, those values were in the same order of magnitude as ours (0.258 mg/kg (Gasparik et al. 2004); 0.21 mg/kg (Santiago et al. 1998)). This indicates long-term exposure to this element. Sewage sludge and mineral fertilizers applied on arable fields are important sources of chemical elements, including toxic ones (Wasi et al. 2013). Indirectly, this generates soil and water acidification, which increases solubility, and hence bioavailability of Cd and Pb for plants (Reglero et al. 2008). The importance of agriculture, as well as forestry, in our study area could therefore be an important source of trace element contamination in the natural environment, including Cd.

In muscle tissue, this element is as low as in bone tissue. Mean Cd content in antlers was low at 0.011 mg/kg. However, it has been shown that chronical exposure to Cd can influence bone tissue posing osteomalacia and osteoporosis (Chmielnicka and Cherian 1986; Wasi et al. 2013).

#### Hg

There are few studies concerning concentrations of Hg in herbivores’ tissues. High Hg concentrations are rarely found in tissues of terrestrial animals, such contamination affecting mainly aquatic organisms. Target organs for this element are kidneys (long-term exposure) and the liver (short-term; Albińska et al. 2011). Higher kidney Hg level than in our study was found in Croatia (0.375 mg/kg) as a result of sediments and mud deposition on plants caused by flooding (Lazarus et al. 2005). Mercury is bound in the soil in insoluble compounds and is poorly absorbed by plants.

The highest environmental Hg deposition is present close to sources of emission and usually refers to accumulation in the local food chain (Gnamuš et al. 2000). Nevertheless, in southern Poland with high mining activity (0.018 mg/kg (Dobrowolska and Melosik 2002); 0.022 mg/kg (Szkoda and Żmudzki 2001); 0.012–0.022 mg/kg (Szkoda et al. 2012)), as well as in Croatia (0.03 mg/kg (Lazarus et al. 2008); 0.011–0.018 mg/kg (Srebočan et al. 2012)), kidney Hg levels were lower than in our study. Conversely, kidney Hg levels were higher in industrial region in eastern Poland (Albińska et al. 2011) and mining area in Spain (Berzas Nevado et al. 2012). This shows the importance of bioavailability of element compound as well as differences among individuals of the same species.

Low Hg concentration (0.001 mg/kg) has been found in muscle tissue, even in animals collected in industrialized, mining areas in Poland (Szkoda and Żmudzki 2001; Szkoda et al. 2012), Spain (Berzas Nevado et al. 2012), and Croatia (Srebočan et al. 2012). Although Hg poorly accumulates in bone tissues, it was detected in all 14 antler samples.

In general, Hg levels in red deer demonstrate low exposure of free-living animals. Nevertheless, concentrations of Hg exceeding the maximum levels in three liver samples and one kidney sample (Table 2; EC 2008) of three specimens indicate individual variations in metal absorption and excretion.

#### As

The highest As level was found in antlers. Chemical similarity of arsenic to phosphorus compounds may explain the accumulation of As in bone tissues (Sharma et al. 2014). Higher As concentrations than in our study were obtained in liver of red deer from both mining and control areas in Spain (0.034–0.061 mg/kg d.w.; Reglero et al. 2008, 2009). In Poland, especially in agricultural areas, use of pesticides and fertilizers represents the main source of As. Uptake of As by plant is generally low and depends on its compounds solubility, soil chemical composition, and vegetation species (Stankovic et al. 2014).

Analysis of As concentrations in red deer tissues collected as nationally representatives samples in Poland were low, 0.016 mg/kg in the liver, 0.005 mg/kg in kidney, and 0.001 in muscles (Szkoda and Żmudzki 2001). These values are well below the threshold recommended by European Food Safety Authority (EFSA 2009). We found 10 times lower liver As level than the mean value reported for deer elsewhere in Europe (0.02 mg/kg w.w.; Frøslie et al. 2001).

### Essential trace elements (Cu, Zn, Fe)

Copper, as Zn and Fe, is an essential trace element, and its status in organisms is restrictively regulated. The highest Cu concentration is found in the liver. In Mazury, mean liver Cu contents were 7.29 mg/kg (this work), 15 mg/kg (Falandysz et al. 2005), and 59 mg/kg d.w. (Jarzyńska and Falandysz 2011). Higher liver Cu concentrations were found in Norway (26.0 mg/kg; Vikøren et al. 2005), Croatia (14.7 mg/kg; Lazarus et al. 2008), Slovenia (13.342 mg/kg; Gasparik et al. 2004), and Spain (35.8–61.4 mg/kg d.w.), where comparison of Cu and Zn levels between contaminated and control regions showed no difference (Reglero et al. 2008). However, Cu values obtained in all studies from Europe were within the range given by Frøslie et al. (2001) for roe deer and red deer (4–30 mg/kg w.w.). Such a wide range of Cu concentrations shows individual variability and various demands of different organisms. Nevertheless, no individual showed visual sympthoms of Cu deficiency.

Unlike Cu, Zn is easily absorbed by plants, which is the main source of this trace element for herbivores. Water contaminated with sewage or wastewater can also be an important source of Zn. Soil chemical analysis in 2005–2007 in NE Poland did not indicate Cu and Zn contamination (Terelak et al. 2008).

The highest Zn levels were found in antlers. This is consistent with Zn requirement for structure and function of alkaline phosphatase, an enzyme involved in bone mineralization. Liver Zn values obtained in this study were much lower than in cervids from unpolluted sites (70–113 mg/kg d.w.; Reglero et al. 2009), and kidney Zn level was in range reported by Frøslie et al. (2001). Surprisingly low concentration of Zn was found in red deer tissues in NW Poland (liver 11.62 mg/kg, kidney 8.52 mg/kg; Wieczorek-Dąbrowska et al. 2013). It is worth to mention that there are much higher levels of Cu and Zn in liver and kidney of red deer from Ontario, Canada (Parker and Hamr 2001). That might be caused by differences in diet and presence of direct pollution source. Nevertheless, authors assessed their values as physiologically correct. Falandysz et al. (2005) reported liver and kidney levels of Cu (1 and 5 mg/kg, respectively) and Zn (30 and 31 mg/kg, respectively) in red deer collected in the same region and stated as physiological levels. Levels of essential trace elements in wildlife should be interpreted locally, as an effect of adaptation to their environmental concentrations. Individual physiological state, sex, and age should be taken into account as well.

Different Zn levels were detected in red deer muscle from the same area, 22.33 mg/kg (Skibniewski et al. 2015), 40 mg/kg (Falandysz et al. 2005), 67.14 mg/kg (this study), and 150 mg/kg d.w. (Jarzyńska and Falandysz 2011). Nevertheless, red deer meat and offal is a good source of Cu and Zn, as human daily demand for those elements is estimated to be 5 mg Cu and 10 mg Zn (Skibniewski et al. 2015).

Absorbed Fe is bound to transferrin. The amount of Fe which is not used directly for hemoglobin production is stored in the liver and bone marrow. Szkoda et al. (2004) reported that Fe concentrations of wild animals across Poland ranged from 3.64 to 170.3 mg/kg in the liver, from 51.81 to 200 mg/kg in the kidney, and from 5.15 to 70.08 mg/kg w.w. in the muscle tissue. Our results are within these ranges for each tissue as well as the data obtained in Croatia (Lazarus et al. 2005, 2008) and Canada (Parker and Hamr 2001). Lower kidney Fe was found in NW Poland (28.76 mg/kg; Wieczorek-Dąbrowska et al. 2013).

Concentration of essential trace elements obtained in our study of free-living red deer and comparison with the literature show great individual variation in this species. Lack of visual sympthoms of deficiency or intoxication indicates efficient mechanisms of homeostasis. Research projects in the same area on different species of wild animals (Falandysz et al. 2005; Giżejewska et al. 2015), soil samples (Terelak et al. 2008), and vegetation (Zalewski and Pacholska 2010) indicate that Mazury has low trace element contaminations.

### Conclusion

Concentrations of Pb, Cd, Hg, As, Cu, Zn, and Fe in red deer antlers cannot be used to predict contaminations of soft tissues. However, we caution that the limited sample size in the current study does not allow for strong inference, and that further research might be warranted with larger sample size, and in different geographic areas. The need to directly analyze trace elements in soft tissues therefore remains to quantitatively assess potential levels of exposure in humans through consumption of venison. Anthropogenic pollution, areas naturally enriched in toxic trace elements, as well as increasing consumption of venison should be reasons to reconsider EC regulations (Lehel et al. 2016). Additionally, studies on Hg and As speciation are necessarily with regard to potential important implication for human health (Ropero et al. 2016). Comparison to levels recommended for livestock tissue consumption indicates that consumption of red deer offal should be limited according to possible contaminations we documented, especially for children, pregnant, and nursing women (Meltzer et al. 2013). Nevertheless, red deer meat is a good source of Cu, Zn, and Fe. Our results confirm that Mazury is little contaminated; however, concentrations of toxic elements in red deer tissues obtained from putatively unpolluted area call for regular monitoring of environmental pollutants.

## Electronic supplementary material

Table S1 (DOC 37 kb)
