# Supplementary material for: Can red deer antlers be used as an indicator of environmental and edible tissues’ trace element contamination?
Source: Environ Sci Pollut Res Int. 2017 Mar 21;24(12):11630–8. doi: 10.1007/s11356-017-8798-7 (PMC5391388; doi:10.1007/s11356-017-8798-7)
Supplement: Supplementary file 1 — (DOC 37 kb) [file 11356_2017_8798_MOESM1_ESM.doc]

Supplementary material

Table S1 Results of linear regression between concentration of analysed seven elements in antlers and in liver, kidney and muscle of red deer (n=14) collected in 2013-2014, NE Poland.

| Element |  | Liver | | |  | Kidney | | |  | Muscle | | |
| --- | --- | --- | --- | --- | --- | --- | --- | --- | --- | --- | --- | --- |
|  |  | F | *p* | R^2^ |  | F | *p* | R^2^ |  | F | *p* | R^2^ |
| Pb |  | 0.19 | 0 .67 | 0.02 |  | 1.80 | 0.20 | 0.13 |  | 0.01 | 0.92 | <0.01 |
| Cd |  | 0.33 | 0 .56 | 0.03 |  | 0.02 | 0.88 | <0.01 |  | 0.74 | 0.41 | 0.06 |
| Hg |  | 0.07 | 0 .80 | 0.01 |  | 0.65 | 0.44 | 0.05 |  | 0.23 | 0.64 | 0.02 |
| As |  | 0.10 | 0 .76 | 0.01 |  | 0.46 | 0.51 | 0.04 |  | 1.90 | 0.18 | 0.14 |
| Cu |  | 0.01 | 0 .93 | <0.01 |  | 0.09 | 0.77 | 0.01 |  | 18.48 | 0.001 | 0.61 |
| Zn |  | 2.70 | 0 .13 | 0.18 |  | 0.84 | 0.38 | 0.07 |  | 2.67 | 0.13 | 0.18 |
| Fe |  | 4.12 | 0 .07 | 0.20 |  | 2.09 | 0.17 | 0.15 |  | 0.09 | 0.76 | 0.01 |
